# Supplementary material for: The mediating role of systemic inflammation and moderating role of racialization in disparities in incident dementia
Source: Commun Med (Lond). 2024 Jul 13;4:142. doi: 10.1038/s43856-024-00569-w (PMC11246521; doi:10.1038/s43856-024-00569-w)
Supplement: Supplementary file 2 — Supplemental information [file 43856_2024_569_MOESM2_ESM.pdf]

Supplemental Table 1: Distribution of baseline characteristics between included and excluded participants in the United States Retirement Study

Characteristic	Overall<br>N = 8,781[1]	Included<br>N = 6,908[1]	Excluded<br>N = 1,873[1]	p-value[2]
**Cognitive Status**				
Incident dementia	795 (12%)	795 (12%)	0 (NA%)	
Cognitively normal or CIND	6,113 (88%)	6,113 (88%)	0 (NA%)	
**Baseline CRP** (μg/mL)	4.55 (8.14)	4.37 (8.00)	5.23 (8.60)	**<0.001**
**Age** (years)	68.12 (10.38)	67.11 (9.91)	71.84 (11.17)	**<0.001**
**Race**				**<0.001**
Non-Hispanic Black	1,057 (12%)	813 (12%)	244 (13%)	
Hispanic	794 (9.0%)	633 (9.2%)	161 (8.6%)	
Non-Hispanic Other	189 (2.2%)	0 (0%)	189 (10%)	
Non-Hispanic white	6,741 (77%)	5,462 (79%)	1,279 (68%)	
**Sex**				**<0.001**
Female	5,290 (60%)	4,234 (61%)	1,056 (56%)	
Male	3,491 (40%)	2,674 (39%)	817 (44%)	
**Education Category**				**<0.001**
> College	812 (9.2%)	676 (9.8%)	136 (7.3%)	
College/Some	1,575 (18%)	1,297 (19%)	278 (15%)	
HS or <	6,394 (73%)	4,935 (71%)	1,459 (78%)	
**Alcohol** (# drinks/day when drinks)	0.70 (1.41)	0.71 (1.30)	0.67 (1.77)	0.279
**Smoking**				**<0.001**
Current	1,176 (13%)	851 (12%)	325 (17%)	
Former	3,774 (43%)	2,966 (43%)	808 (43%)	
Never	3,831 (44%)	3,091 (45%)	740 (40%)	
**Body Mass Index** (kg/m^2)	28.33 (5.88)	28.50 (5.79)	27.70 (6.15)	**<0.001**
**Chronic Conditions**	2.00 (1.42)	1.88 (1.36)	2.44 (1.56)	**<0.001**
**Wave**				**<0.001**
2006	4,178 (48%)	3,485 (50%)	693 (37%)	
2008	4,603 (52%)	3,423 (50%)	1,180 (63%)	
APOE-ε4				0.646
At least 1	2,373 (27%)	1,859 (27%)	514 (27%)	

**Characteristic**	**Overall** N = 8,781[1]	**Included** N = 6,908[1]	**Excluded** N = 1,873[1]	p-value[2]
No copy	6,408 (73%)	5,049 (73%)	1,359 (73%)	

[1]n (%); Mean (SD)

[2]Pearson's Chi-squared test; One-way ANOVA

CRP: C-reactive protein; *APOE-ε4*: apolipoprotein E *ε4* allele carrier status.

CIND: cognitive impairment non-dementia

Supplemental Table 2: Distribution of baseline sample characteristics by racialized social groups in the Health and Retirement Study, 2006 and 2008

**Characteristic**	**Overall**<br>N = 6,908[1]	**Non-Hispanic Black**<br>N = 813[1]	**Hispanic**<br>N = 633[1]	**Non-Hispanic white**<br>N = 5,462[1]	p-value[2]
**Cognitive Status**					**<0.001**
Incident dementia	795 (12%)	171 (21%)	103 (16%)	521 (9.5%)	
Cognitively normal or CIND	6,113 (88%)	642 (79%)	530 (84%)	4,941 (90%)	
**Baseline CRP** (μg/mL)	4.37 (8.00)	6.45 (10.81)	4.45 (6.70)	4.05 (7.59)	**<0.001**
**CRP Category**					**<0.001**
<25th	1,727 (25%)	147 (18%)	122 (19%)	1,458 (27%)	
≥25th & <50th	1,713 (25%)	143 (18%)	161 (25%)	1,409 (26%)	
≥50th & <75th	1,720 (25%)	200 (25%)	182 (29%)	1,338 (24%)	
≥75th	1,748 (25%)	323 (40%)	168 (27%)	1,257 (23%)	
**Age** (years)	67.11 (9.91)	65.89 (9.40)	64.00 (9.89)	67.65 (9.91)	**<0.001**
**Sex**					**<0.001**
Female	4,234 (61%)	553 (68%)	403 (64%)	3,278 (60%)	
Male	2,674 (39%)	260 (32%)	230 (36%)	2,184 (40%)	
**Educational Category**					**<0.001**
> College	676 (9.8%)	42 (5.2%)	13 (2.1%)	621 (11%)	
College/Some	1,297 (19%)	107 (13%)	53 (8.4%)	1,137 (21%)	
HS or <	4,935 (71%)	664 (82%)	567 (90%)	3,704 (68%)	
**Alcohol Use** (# drinks/day when drinks)	0.71 (1.30)	0.49 (1.22)	0.57 (1.31)	0.75 (1.30)	**<0.001**
**Smoking**					**<0.001**
Current	851 (12%)	140 (17%)	83 (13%)	628 (11%)	
Former	2,966 (43%)	328 (40%)	241 (38%)	2,397 (44%)	
Never	3,091 (45%)	345 (42%)	309 (49%)	2,437 (45%)	
**Body Mass Index** (kg/m^2)	28.50 (5.79)	30.51 (6.72)	29.44 (5.42)	28.10 (5.60)	**<0.001**
**Chronic Conditions**	1.88 (1.36)	2.15 (1.43)	1.72 (1.38)	1.85 (1.34)	**<0.001**
APOE-ε4					**<0.001**
At least 1 copy	1,859 (27%)	290 (36%)	137 (22%)	1,432 (26%)	
No copy	5,049 (73%)	523 (64%)	496 (78%)	4,030 (74%)	
**Time**					**<0.001**

Characteristic	Overall<br>N = 6,908[1]	Non-Hispanic Black<br>N = 813[1]	Hispanic<br>N = 633[1]	Non-Hispanic white<br>N = 5,462[1]	p-value[2]
2	302 (4.4%)	80 (9.8%)	39 (6.2%)	183 (3.4%)	
4	269 (3.9%)	52 (6.4%)	37 (5.8%)	180 (3.3%)	
6	6,337 (92%)	681 (84%)	557 (88%)	5,099 (93%)	
**Wave**					**0.004**
2006	3,485 (50%)	377 (46%)	297 (47%)	2,811 (51%)	
2008	3,423 (50%)	436 (54%)	336 (53%)	2,651 (49%)	

[1]n (%); Mean (SD)

[2]Pearson's Chi-squared test; One-way ANOVA

CRP: C-reactive protein; *APOE-ε4*: apolipoprotein E *ε4* allele carrier status.

CIND: cognitive impairment non-dementia

Supplemental Table 3: Baseline levels of C-reactive protein (CRP) by racialized social groups and sex in the United States Health and Retirement Study, waves 2006 & 2008

			C-reactive protein (μg/mL) percentiles							
**Race/ethnicity**	**Sex**	**Observations**	**Mean**	**SD**	**Min**	**Max**	**25th**	**50th**	**75th**	**IQR**
**Non-Hispanic Black**	**Female**	553	6.83	9.99	0.04	85.75	1.41	3.93	8.29	6.88
**Non-Hispanic Black**	**Male**	260	5.63	12.36	0.03	125.05	1.12	2.49	5.84	4.72
**Hispanic**	**Female**	403	4.76	6.48	0.08	78.11	1.47	2.67	5.69	4.22
**Hispanic**	**Male**	230	3.91	7.10	0.04	66.70	0.93	1.88	3.89	2.96
**Non-Hispanic white**	**Female**	3278	4.46	8.10	0.04	172.78	1.02	2.22	5.06	4.04
**Non-Hispanic white**	**Male**	2184	3.43	6.69	0.02	121.10	0.80	1.56	3.44	2.64

Supplemental Table 4: Distribution of baseline sample characteristics by percentiles of C-reactive protein, United States Health and Retirement Study, 2006 and 2008

Characteristic	Overall	C-reactive protein (μg/mL) percentiles				
		**25th (<0.98)**	**25th - 50th (≥0.98 & <2.06)**	**50th - 75th (≥2.06 & <4.73)**	**75th (≥ 4.73)**	
	N = 6,908[1]	N = 1,727[1]	N = 1,713[1]	N = 1,720[1]	N = 1,748[1]	p-value[2]
**Cognitive Status**						0.418
Incident dementia	795 (12%)	199 (12%)	185 (11%)	192 (11%)	219 (13%)	
Cognitively normal or CIND	6,113 (88%)	1,528 (88%)	1,528 (89%)	1,528 (89%)	1,529 (87%)	
**Baseline CRP** (μg/mL)	4.37 (8.00)	0.54 (0.25)	1.46 (0.29)	3.15 (0.76)	12.21 (12.90)	**<0.001**
**Age** (years)	67.11 (9.91)	67.52 (10.29)	67.61 (9.72)	67.32 (9.86)	66.00 (9.70)	**<0.001**
**Race**						**<0.001**
Non-Hispanic Black	813 (12%)	147 (8.5%)	143 (8.3%)	200 (12%)	323 (18%)	
Hispanic	633 (9.2%)	122 (7.1%)	161 (9.4%)	182 (11%)	168 (9.6%)	
Non-Hispanic white	5,462 (79%)	1,458 (84%)	1,409 (82%)	1,338 (78%)	1,257 (72%)	
**Sex**						**<0.001**
Female	4,234 (61%)	939 (54%)	968 (57%)	1,080 (63%)	1,247 (71%)	
Male	2,674 (39%)	788 (46%)	745 (43%)	640 (37%)	501 (29%)	
**Educational Category**						**<0.001**
> College	676 (9.8%)	258 (15%)	167 (9.7%)	133 (7.7%)	118 (6.8%)	
College/Some	1,297 (19%)	347 (20%)	363 (21%)	309 (18%)	278 (16%)	
HS or <	4,935 (71%)	1,122 (65%)	1,183 (69%)	1,278 (74%)	1,352 (77%)	
**Alcohol Use** (# drinks/day when drinks)	0.71 (1.30)	0.79 (1.33)	0.77 (1.34)	0.68 (1.26)	0.59 (1.25)	**<0.001**
**Smoking**						**<0.001**
Current	851 (12%)	151 (8.7%)	183 (11%)	241 (14%)	276 (16%)	
Former	2,966 (43%)	712 (41%)	779 (45%)	728 (42%)	747 (43%)	
Never	3,091 (45%)	864 (50%)	751 (44%)	751 (44%)	725 (41%)	
**Body Mass Index** (kg/m^2)	28.50 (5.79)	26.01 (4.44)	27.55 (4.69)	28.98 (5.31)	31.43 (6.92)	**<0.001**
**Chronic Conditions**	1.88 (1.36)	1.67 (1.35)	1.75 (1.31)	1.91 (1.32)	2.17 (1.41)	**<0.001**
APOE-ε4						**<0.001**
At least 1 copy	1,859 (27%)	601 (35%)	483 (28%)	415 (24%)	360 (21%)	
No copy	5,049 (73%)	1,126 (65%)	1,230 (72%)	1,305 (76%)	1,388 (79%)	

Characteristic	Overall N = 6,908[1]	C-reactive protein (μg/mL) percentiles 25th (<0.98) N = 1,727[1]	25th - 50th (≥0.98 & <2.06) N = 1,713[1]	50th - 75th (≥2.06 & <4.73) N = 1,720[1]	75th (≥ 4.73) N = 1,748[1]	p-value[2]
**Time**						**0.007**
2	302 (4.4%)	59 (3.4%)	84 (4.9%)	64 (3.7%)	95 (5.4%)	
4	269 (3.9%)	72 (4.2%)	53 (3.1%)	81 (4.7%)	63 (3.6%)	
6	6,337 (92%)	1,596 (92%)	1,576 (92%)	1,575 (92%)	1,590 (91%)	
**Wave**						**0.035**
2006	3,485 (50%)	899 (52%)	832 (49%)	839 (49%)	915 (52%)	
2008	3,423 (50%)	828 (48%)	881 (51%)	881 (51%)	833 (48%)	

[1]n (%); Mean (SD)

[2]Pearson's Chi-squared test; One-way ANOVA

CRP: C-reactive protein; *APOE-ε4*: apolipoprotein E *ε4* allele carrier status.

CIND: cognitive impairment non-dementia

Supplemental Table 5: Odds ratio of elevated levels of C-reactive protein (≥4.73μg/mL or ≥ 75th percentile) stratified by minoritized status and racialized social groups in the US Health and Retirement Study, waves 2006 & 2008.

	**Non-Hispanic Black vs non-Hispanic white[1]** N = 6,275		**Hispanic vs non-Hispanic white[1]** N = 6,095		**Minoritized Racial Group vs non-Hispanic white[2]** N = 6,908	
**Models**						
**Unadjusted**	2.21***	[1.89,2.57]	1.21*	[1.00,1.46]	1.72***	[1.52,1.95]
**Demographic[+]**	2.12***	[1.81,2.48]	1.02	[0.84,1.24]	1.58***	[1.38,1.80]
**Risk Factors[$]**	1.72***	[1.44,2.04]	0.99	[0.80,1.21]	1.37***	[1.19,1.58]
**Chronic Conditions[§]**	1.70***	[1.43,2.02]	1.00	[0.81,1.23]	1.37***	[1.19,1.58]

+

Demographic model: adjusted for age, sex, education categories, *APOE-ε4* allele status, and wave.
[$] Risk factors model: adjusted for age, sex, education categories, *APOE-ε4* allele status, wave, smoking status, alcohol consumption, body mass index.
[§] Chronic conditions model: adjusted for age, sex, education categories, *APOE-ε4* allele status, wave, smoking status, alcohol consumption, body mass index, and chronic conditions
[1] Odds ratios, CI: confidence interval in brackets
[2] Odds ratios, CI: confidence intervals in brackets. Minoritized racial group: (non-Hispanic Black and Hispanic participants)
[*] $p < 0.05$, [**] $p < 0.01$, [***] $p < 0.001$

Supplemental Table 6: Randomized analogue mediation models for racial disparities in incident dementia using elevated levels of C-reactive protein (CRP ≥4.73μg/mL) as mediator. Models are stratified by minoritized status and racialized social groups in a sample of United States adults in the Health and Retirement Study

	Randomized Analogue Model+ (Outcome: Incident Dementia)											
	Minoritized Racial Group vs non-Hispanic white N = 6,908				non-Hispanic Black vs non-Hispanic white N = 6,275				Hispanic vs non-Hispanic white N = 6,095			
**Mediator (CRP)**	**Estimate**	**[95%CI]**	**[95%CI]**	**p-value**	**Estimate**	**[95%CI]**	**[95%CI]**	**p-value**	**Estimate**	**[95%CI]**	**[95%CI]**	**p-value**
**Excess Risk**												
RERI Controlled Direct Effect	1.29	0.94	1.70	<0.00	1.67	1.23	2.33	<0.00	0.90	0.45	1.47	<0.00
RERI Interaction Reference	0.17	0.02	0.38	0.04	0.06	-0.16	0.32	0.66	0.38	0.11	0.82	0.01
RERI Interaction Mediation	0.06	0.00	0.09	0.04	0.03	-0.06	0.12	0.65	0.01	-0.09	0.07	0.94
RERI Pure Indirect Effect	0.01	0.00	0.02	0.23	0.01	0.00	0.03	0.14	0.00	-0.01	0.01	0.99
**% Attributable**												
% Controlled Direct Effect	0.84	0.70	0.98	<0.00	0.94	0.76	1.12	<0.00	0.70	0.43	0.91	<0.00
% Interaction Reference	0.11	0.01	0.25	0.04	0.03	-0.09	0.17	0.66	0.30	0.09	0.58	0.01
% Interaction Mediation	0.04	0.00	0.06	0.04	0.02	-0.03	0.06	0.65	0.01	-0.07	0.06	0.94
% Pure Indirect Effect	0.01	0.00	0.01	0.23	0.01	0.00	0.02	0.14	0.00	-0.01	0.01	0.99
Percent Mediated	0.04	0.00	0.06	0.01	0.02	-0.03	0.07	0.42	0.01	-0.08	0.06	0.94
Percent due to Interaction	0.15	0.01	0.29	0.04	0.05	-0.12	0.23	0.66	0.30	0.09	0.57	0.01
Percent Eliminated	0.16	0.02	0.30	0.03	0.06	-0.12	0.24	0.56	0.30	0.09	0.57	0.01

+Model: outcome 6-year incident dementia, model adjusting for age, sex, education categories, wave, smoking status, alcohol consumption, body mass index, alcohol consumption, and chronic conditions, using *APOE-ε4* as variable affected by the racialized social group through ancestry. Minoritized racial group: (non-Hispanic Black and Hispanic participants)

Supplemental Table 7A: Mediational E-values for regression-based models; estimates are presented in the rate ratio scale. Models are stratified by racialized social groups in a sample of the United States adults in the Health and Retirement Study.

	Minoritized vs non-Hispanic white*					
**Rate Ratio Scale**	**Estimate**	**LB 95%CI**	**UB 95%CI**	**E-value**	**E-value LB**	**E-value UB**
Controlled direct effect (Rcde)	2.30	1.94	2.74	4.03	3.28	NA
Pure natural direct effect (Rpnde)	2.43	2.11	2.81	4.29	3.64	NA
Total natural direct effect (Rtnde)	2.46	2.13	2.83	4.35	3.69	NA
Pure natural indirect effect (Rpnie)	1.01	1.00	1.02	1.09	1.00	NA
Total natural indirect effect (Rtnie)	1.02	1.00	1.04	1.16	1.06	NA
Total effect (Rte)	2.47	2.15	2.85	4.38	3.72	NA
	**non-Hispanic Black vs non-Hispanic white***					
	**Estimate**	**LB 95%CI**	**UB 95%CI**	**E-value**	**E-value LB**	**E-value UB**
Controlled direct effect (Rcde)	2.65	2.18	3.30	4.75	3.79	NA
Pure natural direct effect (Rpnde)	2.65	2.25	3.20	4.75	3.92	NA
Total natural direct effect (Rtnde)	2.65	2.26	3.20	4.75	3.95	NA
Pure natural indirect effect (Rpnie)	1.01	1.00	1.04	1.11	1.00	NA
Total natural indirect effect (Rtnie)	1.01	0.98	1.05	1.11	1.00	NA
Total effect (Rte)	2.68	2.29	3.22	4.80	4.00	NA
	**Hispanic vs non-Hispanic white***					
	**Estimate**	**LB 95%CI**	**UB 95%CI**	**E-value**	**E-value LB**	**E-value UB**
Controlled direct effect (Rcde)	1.98	1.49	2.58	3.36	2.34	NA
Pure natural direct effect (Rpnde)	2.33	1.86	2.86	4.09	3.13	NA
Total natural direct effect (Rtnde)	2.31	1.85	2.85	4.05	3.11	NA
Pure natural indirect effect (Rpnie)	1.00	0.99	1.01	1.05	NA	1.00
Total natural indirect effect (Rtnie)	0.99	0.96	1.03	1.11	NA	1.00
Total effect (Rte)	2.31	1.85	2.84	4.04	3.10	NA

* These E-values are on the rate ratio scale and correspond to the decomposition presented on Table 3
LB: Lower bound; UB: Upper bound
95%CI: 95% Confidence interval
NA: not applicable

Supplemental Table 7B: Mediational E-values for randomized analogue models; estimates are presented in the rate ratio scale. Models are stratified by racialized social groups in a sample of the United States adults in the Health and Retirement Study.

	Minoritized vs non-Hispanic white*					
**Rate Ratio Scale**	**Estimate**	**LB 95%CI**	**UB 95%CI**	**E-value**	**E-value LB**	**E-value UB**
Controlled direct effect (Rcde)	2.33	1.97	2.78	4.08	3.35	NA
Pure natural direct effect (rRpnde)+	2.46	2.15	2.88	4.36	3.73	NA
Total natural direct effect (rRtnde)+	2.50	2.17	2.92	4.45	3.77	NA
Pure natural indirect effect (rRpnie)+	1.01	1.00	1.02	1.11	1.00	NA
Total natural indirect effect (rRtnie)+	1.03	1.00	1.04	1.19	1.04	NA
Total effect (Rte)	2.53	2.19	2.93	4.49	3.80	NA
	**non-Hispanic Black vs non-Hispanic white***					
	**Estimate**	**LB 95%CI**	**UB 95%CI**	**E-value**	**E-value LB**	**E-value UB**
Controlled direct effect (Rcde)	2.73	2.29	3.43	4.91	4.00	NA
Pure natural direct effect (rRpnde)+	2.73	2.35	3.34	4.91	4.13	NA
Total natural direct effect (rRtnde)+	2.74	2.35	3.29	4.91	4.14	NA
Pure natural indirect effect (rRpnie)+	1.01	1.00	1.03	1.13	1.00	NA
Total natural indirect effect (rRtnie)+	1.02	0.98	1.05	1.14	1.00	NA
Total effect (Rte)	2.77	2.38	3.35	4.99	4.19	NA
	**Hispanic vs non-Hispanic white***					
	**Estimate**	**LB 95%CI**	**UB 95%CI**	**E-value**	**E-value LB**	**E-value UB**
Controlled direct effect (Rcde)	1.93	1.46	2.53	3.27	2.28	NA
Pure natural direct effect (rRpnde)+	2.28	1.87	2.85	3.99	3.15	NA
Total natural direct effect (rRtnde)+	2.29	1.87	2.83	4.01	3.15	NA
Pure natural indirect effect (rRpnie)+	1.00	0.99	1.01	1.02	1.00	NA
Total natural indirect effect (rRtnie)+	1.00	0.96	1.04	1.07	1.00	NA
Total effect (Rte)	2.29	1.87	2.84	4.01	3.15	NA

* These E-values are on the rate ratio scale and correspond to the decomposition presented on Supplemental Table 6
+ Randomized analogue estimate
LB: Lower bound; UB: Upper bound
95%CI: 95% Confidence interval
NA: not applicable

Supplemental Table 8: Randomized analogue mediation models for racial disparities in incident dementia using elevated levels of C-reactive protein (CRP ≥4.73μg/mL) as mediator. Models are stratified by minoritized status and racialized social groups in a sample of United States adults in the Health and Retirement Study

	Randomized Analogue Model+ (Outcome: Incident Dementia)											
	Minoritized Racial Group vs non-Hispanic white N = 6,908				non-Hispanic Black vs non-Hispanic white N = 6,275				Hispanic vs non-Hispanic white N = 6,095			
**Mediator (CRP)**	**Estimate**	**[95%CI]**	**[95%CI]**	**p-value**	**Estimate**	**[95%CI]**	**[95%CI]**	**p-value**	**Estimate**	**[95%CI]**	**[95%CI]**	**p-value**
**Excess Risk**												
RERI Controlled Direct Effect	1.29	0.91	1.70	<0.00	1.69	1.20	2.27	<0.00	0.90	0.43	1.46	0.00
RERI Interaction Reference	0.19	0.02	0.39	0.03	0.07	-0.15	0.35	0.55	0.41	0.11	0.82	0.00
RERI Interaction Mediation	0.05	0.00	0.09	0.03	0.02	-0.05	0.11	0.55	-0.02	-0.10	0.07	0.94
RERI Pure Indirect Effect	0.01	0.00	0.02	0.23	0.01	0.00	0.03	0.15	0.00	-0.01	0.01	0.96
**% Attributable**												
% Controlled Direct Effect	0.84	0.71	0.98	<0.00	0.95	0.75	1.11	0.00	0.70	0.44	0.91	0.00
% Interaction Reference	0.12	0.01	0.25	0.03	0.04	-0.09	0.18	0.55	0.32	0.09	0.57	0.00
% Interaction Mediation	0.03	0.00	0.06	0.03	0.01	-0.03	0.06	0.55	-0.02	-0.07	0.05	0.94
% Pure Indirect Effect	0.00	0.00	0.01	0.23	0.00	0.00	0.02	0.15	0.00	-0.01	0.01	0.96
Percent Mediated	0.03	0.00	0.06	0.01	0.01	-0.02	0.07	0.35	-0.02	-0.08	0.06	0.94
Percent due to Interaction	0.15	0.02	0.28	0.03	0.05	-0.12	0.25	0.55	0.30	0.09	0.56	0.00
Percent Eliminated	0.16	0.02	0.29	0.02	0.05	-0.11	0.25	0.47	0.30	0.09	0.56	0.00

+Model: outcome 6-year incident cognitive impairment, model adjusting for age, sex, wave, smoking status, alcohol consumption, body mass index, alcohol consumption, and chronic conditions, using *APOE-ε4* and educational attainment as variables affected by racialized social group. Minoritized racial group: (non-Hispanic Black and Hispanic participants)

Supplemental Table 9: Incidence rate ratios from Poisson regression analysis, estimates represent the association between elevated levels of C-reactive protein (CRP) ($\geq$4.73µg/mL) and 6-year cognitive impairment (dementia or cognitive impairment non-dementia) in the United States Health and Retirement Study. Models are stratified by racialized social groups and minoritized status.

	Incident Cognitive Impairment (dementia or cognitive impairment non-dementia)									
	**Overall** N = 6,151		**Minoritized** (non-Hispanic Black & Hispanic) N = 1,065		**Non-Hispanic Black** N = 580		**Hispanic** N = 485		**Non-Hispanic white** N = 5,086	
**Models**										
**Unadjusted**	**IRR[1]**	**95% CI[1]**	**IRR[1]**	**95% CI[1]**	**IRR[1]**	**95% CI[1]**	**IRR[1]**	**95% CI[1]**	**IRR[1]**	**95% CI[1]**
< 75th	1	-	1	-	1	-	1	-	1	-
$\geq$ 75th ($\geq$4.73µg/mL)	1.06	[0.96,1.18]	1.02	[0.86,1.22]	0.97	[0.77,1.21]	1.10	[0.82,1.46]	1.00	[0.88,1.13]
**Demographic⁺**										
< 75th	1	-	1	-	1	-	1	-	1	-
$\geq$ 75th ($\geq$4.73µg/mL)	1.08	[0.97,1.19]	1.04	[0.87,1.24]	1.00	[0.80,1.26]	1.08	[0.81,1.43]	1.08	[0.96,1.22]
**Risk Factors$**										
< 75th	1	-	1	-	1	-	1	-	1	-
$\geq$ 75th ($\geq$4.73µg/mL)	1.07	[0.96,1.19]	1.04	[0.86,1.25]	1.01	[0.80,1.29]	1.07	[0.79,1.44]	1.08	[0.95,1.22]
**Chronic Conditions§**										
< 75th	1	-	1	-	1	-	1	-	1	-
$\geq$ 75th ($\geq$4.73µg/mL)	1.05	[0.95,1.17]	1.02	[0.85,1.23]	1.01	[0.80,1.28]	1.07	[0.80,1.43]	1.05	[0.93,1.19]

[+] Demographic model: adjusted for age, sex, education categories, *APOE-ε4* allele status, and wave. Note: the demographic model in the overall sample (N=6,151) additionally adjust for racialized social groups

[$] Risk factors model: adjusted for age, sex, education categories, *APOE-ε4* allele status, wave, smoking status, alcohol consumption, body mass index.

[§] Chronic conditions model: adjusted for age, sex, education categories, *APOE-ε4* allele status, wave, smoking status, alcohol consumption, body mass index, and chronic conditions

[1] IRR: incidence rate ratio, CI: confidence interval in brackets,

* $p < 0.05$, ** $p < 0.01$, *** $p < 0.001$

Supplemental Table 10: Incidence rate ratios from Poisson regression analysis, estimates represent the association between C-reactive protein (CRP) (standardized log transformation) and 6-year incident dementia in the United States Health and Retirement Study. Models are stratified by racialized social groups and minoritized status.

	Incident Dementia									
	**Overall** N = 6,908		**Minoritized** (non-Hispanic Black & Hispanic) N = 1,446		**Non-Hispanic Black** N = 813		**Hispanic** N = 633		**Non-Hispanic white** N = 5,462	
**Models**										
**Unadjusted**	**IRR[1]**	**95% CI[1]**	**IRR[1]**	**95% CI[1]**	**IRR[1]**	**95% CI[1]**	**IRR[1]**	**95% CI[1]**	**IRR[1]**	**95% CI[1]**
Log CRP	1.04	[0.96,1.11]	0.99	[0.88,1.12]	0.95	[0.82,1.09]	1.05	[0.86,1.30]	1.00	[0.91,1.09]
**Demographic[+]**										
Log CRP	1.05	[0.98,1.13]	1.05	[0.94,1.17]	1.01	[0.88,1.16]	1.09	[0.89,1.33]	1.05	[0.96,1.15]
**Risk Factors[$]**										
Log CRP	1.07	[0.99,1.15]	1.05	[0.93,1.18]	1.02	[0.88,1.18]	1.07	[0.98,1.17]	1.07	[0.98,1.17]
**Chronic Conditions[§]**										
Log CRP	1.06	[0.99,1.14]	1.05	[0.93,1.19]	1.03	[0.89,1.19]	1.03	[0.89,1.16]	1.07	[0.98,1.16]

[+] Demographic model: adjusted for age, sex, education categories, *APOE-ε4* allele status, and wave. Note: the demographic model in the overall sample (N=6,908) additionally adjust for racialized social groups

[$] Risk factors model: adjusted for age, sex, education categories, *APOE-ε4* allele status, wave, smoking status, alcohol consumption, body mass index.

[§] Chronic conditions model: adjusted for age, sex, education categories, *APOE-ε4* allele status, wave, smoking status, alcohol consumption, body mass index, and chronic conditions

[1] IRR: incidence rate ratio, CI: confidence interval in brackets,

* $p < 0.05$, ** $p < 0.01$, *** $p < 0.001$

Supplemental Table 11: 4-way mediation analysis decomposition for racialized disparities in incident dementia using C-reactive protein (standardized log transformation) as mediator. Models are stratified by minoritized status and racialized social groups in a sample of United States adults in the Health and Retirement Study

	Incident Dementia*											
	Minoritized Racial Group vs non-Hispanic white N = 6,908				non-Hispanic Black vs non-Hispanic white N = 6,275				Hispanic vs non-Hispanic white N = 6,095			
**Mediator (CRP)**	**Estimate**	**[95%CI]**	**[95%CI]**	**p-value**	**Estimate**	**[95%CI]**	**[95%CI]**	**p-value**	**Estimate**	**[95%CI]**	**[95%CI]**	**p-value**
**Excess Risk**												
RERI Controlled Direct Effect	1.45	1.12	1.84	0.00	1.65	1.23	2.15	0.00	1.30	0.84	1.83	0.00
RERI Interaction Reference	0.00	-0.02	0.03	0.62	-0.01	-0.02	0.03	0.63	0.01	-0.01	0.10	0.68
RERI Interaction Mediation	0.03	-0.01	0.08	0.12	0.04	-0.04	0.14	0.29	0.01	-0.01	0.07	0.38
RERI Pure Indirect Effect	0.00	-0.01	0.02	0.40	0.01	-0.01	0.03	0.21	0.00	0.00	0.01	0.46
**% Attributable**												
% Controlled Direct Effect	0.98	0.93	1.00	0.00	0.97	0.91	1.01	0.00	0.98	0.88	1.01	0.00
% Interaction Reference	0.00	-0.01	0.02	0.62	-0.01	-0.01	0.02	0.63	0.01	-0.01	0.08	0.68
% Interaction Mediation	0.02	0.00	0.05	0.12	0.03	-0.02	0.08	0.29	0.01	-0.01	0.05	0.38
% Pure Indirect Effect	0.00	0.00	0.01	0.40	0.01	0.00	0.02	0.21	0.00	0.00	0.01	0.46
Percent Mediated	0.02	0.00	0.05	0.06	0.03	-0.02	0.09	0.19	0.01	-0.01	0.05	0.33
Percent due to Interaction	0.02	0.00	0.06	0.19	0.02	-0.01	0.09	0.33	0.02	-0.01	0.12	0.37
Percent Eliminated	0.02	0.00	0.07	0.06	0.03	-0.01	0.09	0.16	0.02	-0.01	0.12	0.29

*Outcome 6-year incident dementia, model adjusting for age, sex, education categories, *APOE-ε4* allele status, wave, smoking status, alcohol consumption, body mass index, and chronic conditions. Minoritized racial group: (non-Hispanic Black and Hispanic participants)

Supplemental Table 12: Randomized analogue mediation model for racial disparities in incident dementia using C-reactive protein (standardized log transformation) as mediator. Models are stratified by minoritized status and racialized social groups in a sample of United States adults in the Health and Retirement Study

	Incident Dementia+											
	Minoritized Racial Group vs non-Hispanic white N = 6,908				non-Hispanic Black vs non-Hispanic white N = 6,275				Hispanic vs non-Hispanic white N = 6,095			
**Mediator (CRP)**	**Estimate**	**[95%CI]**	**[95%CI]**	**p-value**	**Estimate**	**[95%CI]**	**[95%CI]**	**p-value**	**Estimate**	**[95%CI]**	**[95%CI]**	**p-value**
**Excess Risk**												
RERI Controlled Direct Effect	1.48	1.15	1.89	0.00	1.72	1.32	2.29	0.00	1.25	0.81	1.74	0.00
RERI Interaction Reference	0.01	-0.01	0.06	0.51	0.02	0.00	0.07	0.53	0.03	0.00	0.15	0.28
RERI Interaction Mediation	0.02	-0.01	0.08	0.12	0.03	-0.03	0.13	0.29	0.00	-0.01	0.08	0.35
RERI Pure Indirect Effect	0.00	-0.01	0.02	0.40	0.01	-0.01	0.03	0.22	0.00	0.00	0.01	0.45
**% Attributable**												
% Controlled Direct Effect	0.98	0.92	1.00	0.00	0.97	0.89	1.01	0.00	0.97	0.84	1.00	0.00
% Interaction Reference	0.01	0.00	0.04	0.51	0.01	0.00	0.04	0.53	0.02	0.00	0.12	0.28
% Interaction Mediation	0.02	0.00	0.05	0.12	0.02	-0.02	0.07	0.29	0.00	-0.01	0.06	0.35
% Pure Indirect Effect	0.00	0.00	0.01	0.40	0.00	0.00	0.01	0.22	0.00	0.00	0.01	0.45
Percent Mediated	0.02	0.00	0.05	0.06	0.02	-0.01	0.07	0.19	0.00	-0.01	0.06	0.31
Percent due to Interaction	0.02	-0.01	0.08	0.15	0.03	-0.02	0.10	0.31	0.03	-0.01	0.16	0.22
Percent Eliminated	0.02	0.00	0.08	0.07	0.03	-0.01	0.11	0.20	0.03	0.00	0.16	0.18

+ Model: outcome 6-year incident dementia, model adjusting for age, sex, education categories, wave, smoking status, alcohol consumption, body mass index, and chronic conditions, using *APOE-ε4* allele status as variable affected by the racialized social group through ancestry. Minoritized racial group: (non-Hispanic Black and Hispanic participants)

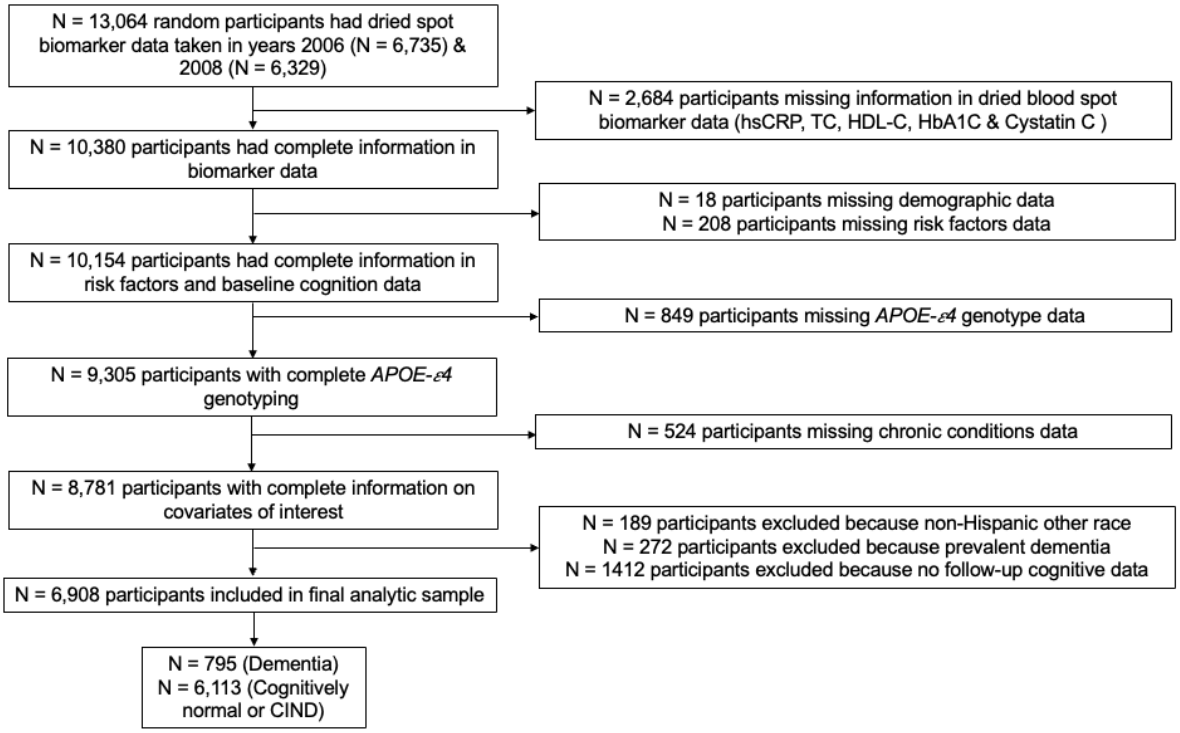

Supplemental Figure 1: Flow diagram of analytic sample study in the Health and Retirement Study. CRP: C-reactive protein, TC: total cholesterol; HDL-C: high density lipoprotein; HbA1C: glycosylated hemoglobin, *APOE-ε4*: apolipoprotein E *ε4* allele carrier status, CIND: cognitive impairment non-dementia.

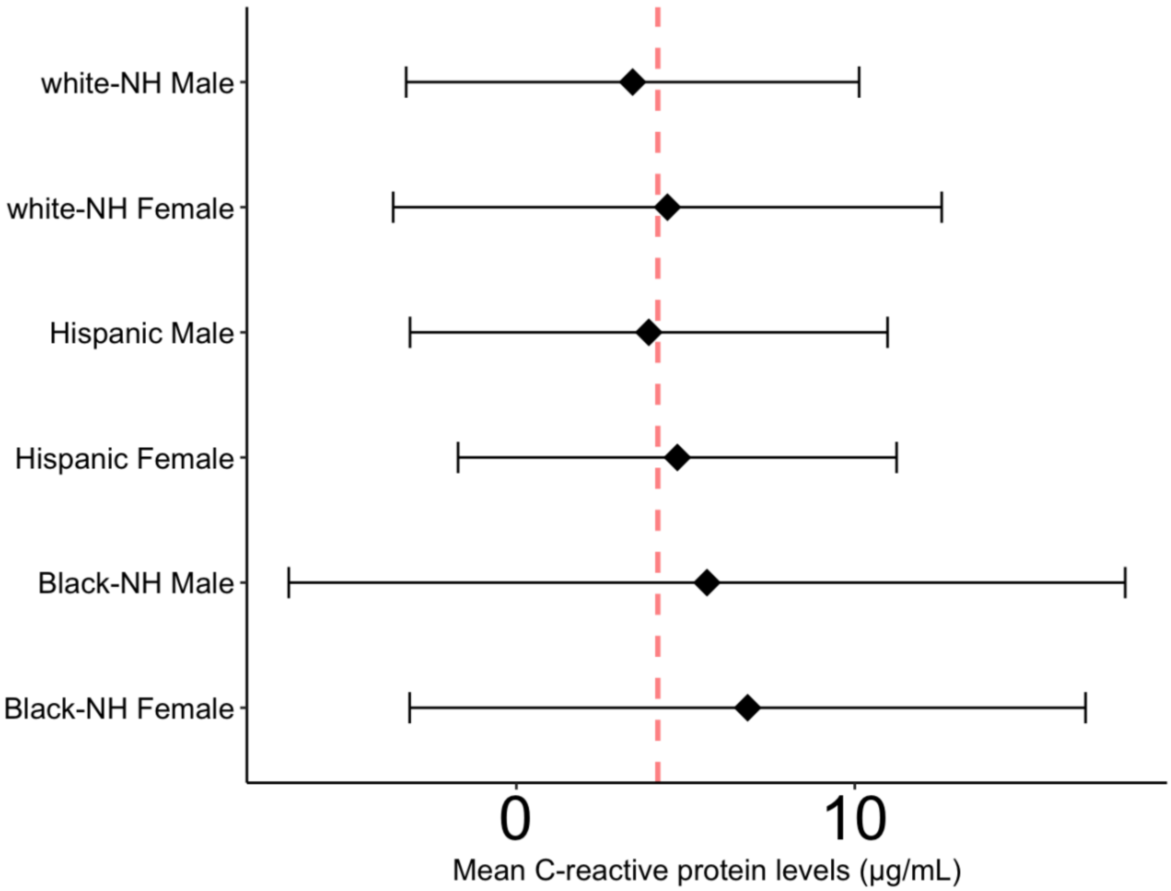

Supplemental Figure 2: Baseline C-reactive protein levels (µg/mL) by racialized social groups and sex in the United States Health and Retirement Study, waves 2006 & 2008. Dotted red line denotes the average levels of C-reactive protein in the overall sample (4.37µg/mL). Dots represent mean C-reactive protein levels in each group and error bars illustrate the standard deviation. white-NH: non-Hispanic white (n=5,462; non-Hispanic white women, n=3,278). Black-NH: non-Hispanic Black (n=813; non-Hispanic Black women, n=553). Hispanic (n=633; Hispanic women = 403)

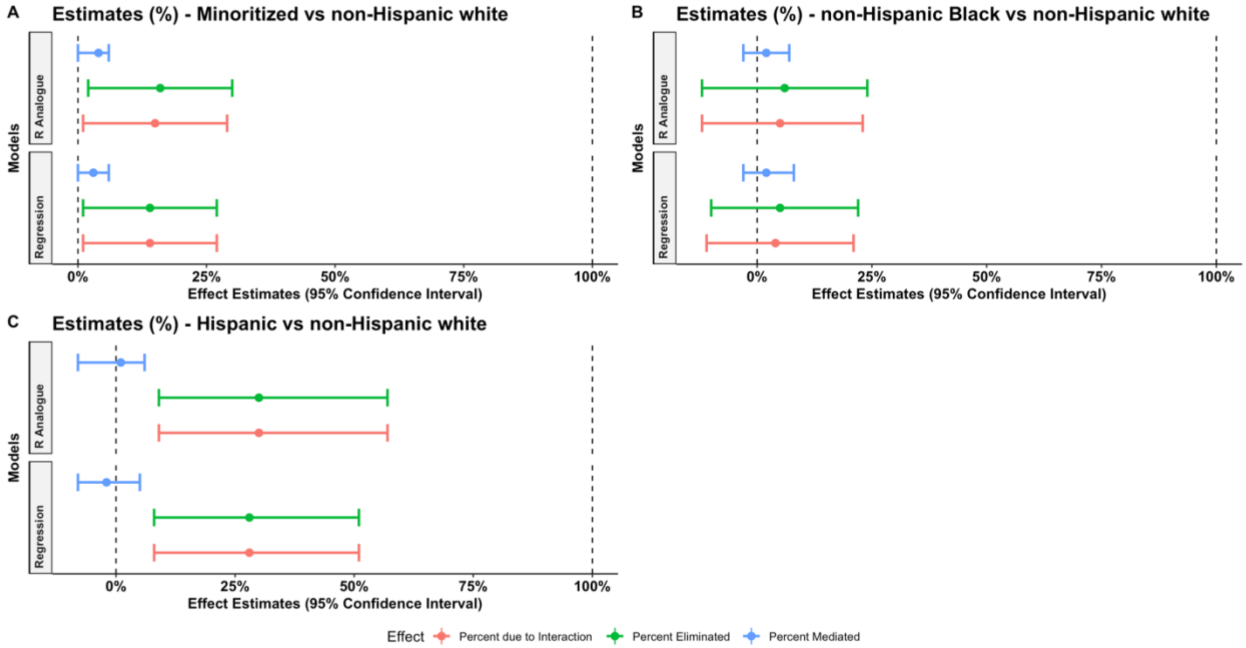

Supplemental Figure 3: Plot of mediation analysis estimates from regression-based and randomized analogue models denoting percent of the racial disparity in incident dementia that is due to the mediating effect of C-reactive protein, the percent due to the interaction between exposure and mediator, and the proportion eliminated. **A.** Mediation estimates from the racial disparity between the minoritized racial group (non-Hispanic Black and Hispanic) vs the non-Hispanic white group. **B.** Mediation estimates from the racial disparity between the non-Hispanic Black vs the non-Hispanic white group. **C.** Mediation estimates from the racial disparity between the Hispanic group vs the non-Hispanic white group.

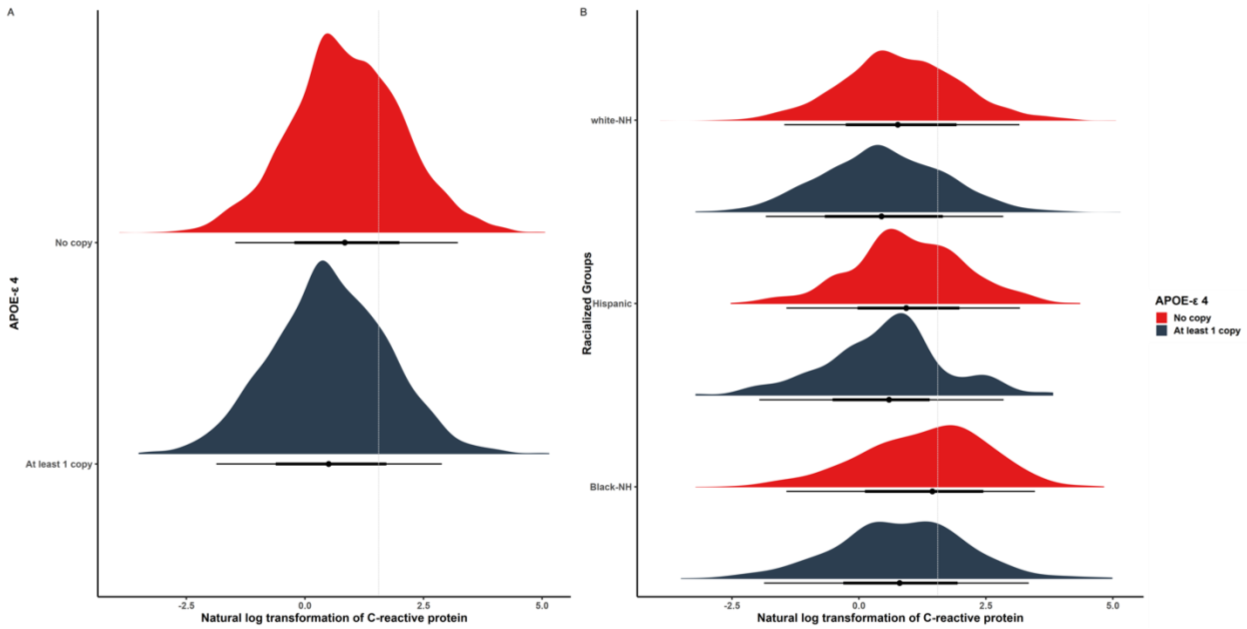

Supplemental Figure 4: Density plot of natural logarithmic transformation of C-reactive protein (CRP) in a selected sample of United States adults in the Health and Retirement Study.
A. Distribution of the natural logarithmic transformation of C-reactive protein (CRP) by *APOE-ε4* allele carrier status in our selected sample from the Health and Retirement Study (HRS). Dotted line denotes the cut off point for elevated levels of CRP at the 75th percentile ($\geq$ 4.73µg/mL, n=8,320). **B.** Distribution of CRP by racialized groups and *APOE-ε4* allele carrier status. white-NH: non-Hispanic white (n=6,602), Black-NH: non-Hispanic Black (n=971), Hispanic (n=747)
