# Supplementary material for: The mediating role of systemic inflammation and moderating role of racialization in disparities in incident dementia
Source: Commun Med (Lond). 2024 Jul 13;4:142. doi: 10.1038/s43856-024-00569-w (PMC11246521; doi:10.1038/s43856-024-00569-w)
Supplement: Supplementary file 3 — Description of Additional Supplementary Files [file 43856_2024_569_MOESM3_ESM.pdf]

Description of Additional Supplementary Files

File name: Supplementary Data 1

File Description: This file is the data source to create the main Figure 2 of the manuscript.
